# Effect of pravastatin on survival in patients with advanced hepatocellular carcinoma. A randomized controlled trial

**DOI:** 10.1054/bjoc.2000.1716

**Published:** 2001-04

**Authors:** S Kawata, E Yamasaki, T Nagase, Y Inui, N Ito, Y Matsuda, M Inada, S Tamura, S Noda, Y Imai, Y Matsuzawa

**Affiliations:** Department of Internal Medicine and Molecular Science, Graduate School of Medicine, Osaka University, Osaka, 565-0871, Japan; Department of Internal Medicine, Yamagata University School of Medicine, Yamagata, 990-9585, Japan

**Keywords:** hepatocellular carcinoma, pravastatin, HMG-CoA reductase inhibitor, survival

## Abstract

Chemotherapy is not effective for hepatocellular carcinoma (HCC). HMG-CoA redutase inhibitors have cytostatic activity for cancer cells, but their clinical usefulness is unknown. To investigate whether pravastatin, a potent HMG-CoA reductase inhibitor, prolongs survival in patients with advanced HCC, this randomized controlled trial was conducted between February 1990 and February 1998 at Osaka University Hospital. 91 consecutive patients <71 years old (mean age 62) with unresectable HCC were enroled in this study. 8 patients were withdrawn because of progressive liver dysfunction; 83 patients were randomized to standard treatment with or without pravastatin. All patients underwent transcatheter arterial embolization (TAE) followed by oral 5-FU 200 mg^−1^d for 2 months. Patients were then randomly assigned to control (*n* = 42) and pravastatin (*n* = 41) groups. Pravastatin was administered at a daily dose of 40 mg. The effect of pravastatin on tumour growth was assessed by ultrasonography. Primary endpoint was death due to progression of HCC. The duration of pravastatin administration was 16.5 ± 9.8 months (mean ± SD). No patients in either group were lost to follow-up. Median survival was 18 months in the pravastatin group versus 9 months in controls (*P* = 0.006). The Cox proportional hazards model showed that pravastatin was a significant factor contributing to survival. Pravastatin prolonged the survival of patients with advanced HCC, suggesting its value for adjuvant treatment. © 2001 Cancer Research Campaign http://www.bjcancer.com


*British Journal of Cancer* (2001) **84**(7), 886–891
© 2001 Cancer Research Campaign
doi: 10.1054/ bjoc.2001.1716, available online at http://www.idealibrary.com on IDEAL®

### Effect of pravastatin on survival in patients with advanced hepatocellular carcinoma. A randomized controlled trial

**S Kawata[1, 2], E Yamasaki[1], T Nagase[1], Y Inui[1], N Ito[1], Y Matsuda[1], M Inada[1], S Tamura[1], S Noda[1], Y Imai[1] and Y Matsuzawa[1]**

[1]Department of Internal Medicine and Molecular Science, Graduate School of Medicine, Osaka University, Osaka 565-0871, Japan, and [2]Department of Internal Medicine, Yamagata University School of Medicine, Yamagata 990-9585, Japan

**Summary** Chemotherapy is not effective for hepatocellular carcinoma (HCC). HMG-CoA redutase inhibitors have cytostatic activity for cancer cells, but their clinical usefulness is unknown. To investigate whether pravastatin, a potent HMG-CoA reductase inhibitor, prolongs survival in patients with advanced HCC, this randomized controlled trial was conducted between February 1990 and February 1998 at Osaka University Hospital. 91 consecutive patients <71 years old (mean age 62) with unresectable HCC were enroled in this study. 8 patients were withdrawn because of progressive liver dysfunction; 83 patients were randomized to standard treatment with or without pravastatin. All patients underwent transcatheter arterial embolization (TAE) followed by oral 5-FU 200 mg$^{-1}$ d for 2 months. Patients were then randomly assigned to control ($n$ = 42) and pravastatin ($n$ = 41) groups. Pravastatin was administered at a daily dose of 40 mg. The effect of pravastatin on tumour growth was assessed by ultrasonography. Primary endpoint was death due to progression of HCC. The duration of pravastatin administration was 16.5 ± 9.8 months (mean ± SD). No patients in either group were lost to follow-up. Median survival was 18 months in the pravastatin group versus 9 months in controls ($P$ = 0.006). The Cox proportional hazards model showed that pravastatin was a significant factor contributing to survival. Pravastatin prolonged the survival of patients with advanced HCC, suggesting its value for adjuvant treatment. © 2001 Cancer Research Campaign http://www.bjcancer.com

**Keywords**: hepatocellular carcinoma; pravastatin; HMG-CoA reductase inhibitor; survival

Hepatocellular carcinoma (HCC) causes death in patients with cirrhosis and is one of the most prevalent malignant tumours worldwide (Simonetti et al, 1991; Okuda, 1992; Di Bisceglie, 1995). Its incidence has substantially increased in Japan (Okuda et al, 1987) and in the United States (El-Serag and Mason, 1999). HCC has a dismal 5-year survival rate, and there is no effective chemotherapy.

Signal transduction inhibitors, including farnesyl transferase inhibitors and mitogen-activated protein kinase (MAPK) kinase inhibitors, have been developed as anti-cancer agents (Gibbs et al, 1993; James et al, 1993; Kohl et al, 1993; Stebolt-Leopold et al, 1999). The activity of 3-hydroxy-3-methylglutaryl coenzyme A (HMG-CoA) reductase, the rate-limiting enzyme of cholesterol biosynthesis, has been positively correlated with mammalian cell growth (Kandutsch and Chen, 1979). Mevalonic acid, produced by HMG-CoA reductase, regulates cell growth independent of cholesterogenesis: Ras p21 and lamins A and B undergo covalent modification at the carboxyl terminus by mevalonate-derived farnesyl isoprenoid (Goldstein and Brown, 1990). HMG-CoA reductase inhibitors exhibit cytostatic activity possibly as signal transduction inhibitors, when added to proliferating cells in culture or in vivo (Goldstein et al, 1979; Habenicht et al, 1980; Maltese et al, 1985). Decreased farnesyl isoprenoid formation by these inhibitors could lead to suppression of tumour growth by interfering with the function of Ras p21. However, there are no report on whether such inhibitors have potential in cancer patients.

In this study, we tested whether administration of HMG-CoA reductase inhibitor would contribute to the survival of patients with advanced HCC. We administered pravastatin (40 mg day$^{-1}$), for which the liver has a high affinity, to HCC patients in a randomized controlled trial after transcatheter arterial embolization (TAE) (Charnsagavej et al, 1983; Yamada et al, 1983; Stefanini et al, 1995) and oral 5-fluorouracil (5-FU) as standard treatment.

### PATIENTS AND METHODS

### Patients

The cohort comprised 91 consecutive patients with unresectable advanced HCC who were younger than 70 years old (Figure 1); 71 patients were male and 20 were female. The mean age was 62 (ranging from 39 to 70). The diagnosis of cirrhosis was confined by biochemical data and ultrasonography (US). The histologic diagnosis of underlying liver disease was carried out in 47 patients by US-guided liver biopsy. The diagnosis of HCC was based on clinical features and findings from US, computed tomography, and hepatic arteriography. Tumour stage (I–IV) was determined according to the criteria of the Primary Liver Cancer Study Group of Japan: stage I, a single tumour ≤2 cm in its greatest dimension without vascular invasion; stage II, a single tumour <2 cm in its greatest dimension with vascular invasion, or multiple tumours

*Received 22 February 2000*
*Revised 28 December 2000*
*Accepted 19 January 2001*

*Correspondence to:* S Kawata

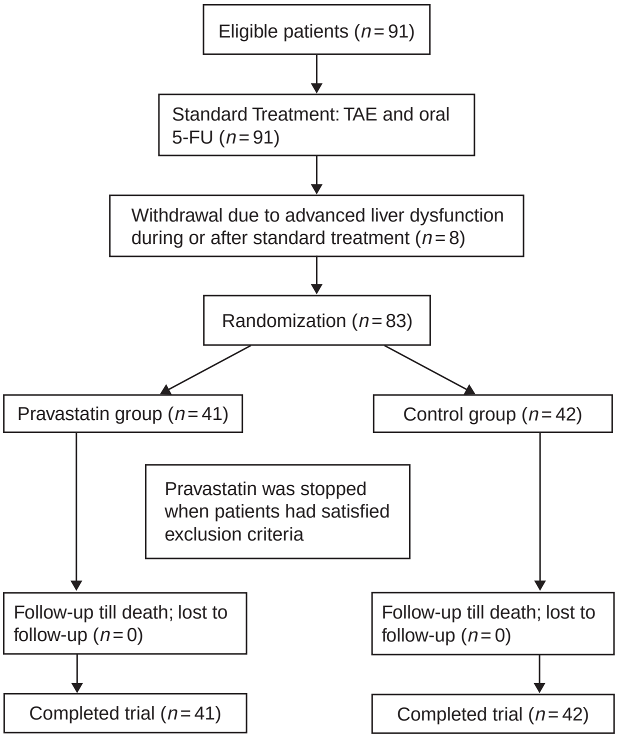

**Figure 1** Protocol for enrolment, randomization and follow-up. TAE; transcatheter arterial embolization with a maximum diameter ≤2 cm confined to one lobe, or a single tumour with a diameter >2 cm, without vascular invasion; stage III, a single tumour with a diameter >2 cm, with vascular invasion, or multiple tumours >2 cm confined to one lobe; stage IV, multiple tumours in more than one lobe or associated vascular invasion in the first branch of the portal or hepatic veins (The Liver Cancer Study Group of Japan, 1989). The histologic diagnosis of HCC was confirmed in the 47 patients with US-guided biopsy. All patients had died by the end of February 1998, and the histologic diagnosis of HCC was confirmed by autopsy or needle necropsy in all patients.

As standard treatment, all patients underwent transcatheter arterial embolization (TAE). For the TAE procedure, patients were treated with gelatin-sponge particles and ethyl ester of poppyseed oil fatty acids containing 38% iodine by weight (Lipiodol; Andre-Gelbe Laboratories, Paris, France) after intraarterial infusion of doxorubicin (30 mg) (Matsuda et al, 1994). Oral 5-FU at a daily dose of 200 mg was started 2 weeks after the TAE procedure and continued for 2 months.

8 of 91 patients were withdrawn during standard treatment for any of the following exclusion criteria: hyperbilirubinaemia >51 micro mol $l^{-1}$; hyperammonaemia >70 micro mol $l^{-1}$; prothrombin time >14 seconds; hypoalbuminaemia <25 g $l^{-1}$; serum alanine aminotransferase (ALT) level >150 U $l^{-1}$; or massive ascites. The remaining 83 patients were randomly divided into control ($n$ = 42) and pravastatin ($n$ = 41) groups after standard treatment; randomization was generated by a computer program. Survival analysis in both groups began on the 15th day after 5-FU was completed. The pravastatin group received oral drug at a dose of 20 mg beginning on the 15th day after the end of 5-FU administration. 2 weeks later, pravastatin was increased to 40 mg per day. The pravastatin group was not treated with any other anti-cancer drugs. Pravastatin was discontinued when patients showed any of the exclusion criteria, or signs and symptoms ascribed to adverse effects of the drug. The control group was not treated with any anti-cancer drugs. Written informed consent was obtained from all subjects prior to entry. The protocol in this study was approved by the local scientific ethical committee.

### Assessment

Death was the primary endpoint. Clinical status and laboratory data, including hepatic and renal function tests and haematologic examinations, were followed at least once a month in the outpatient clinics of Osaka University Hospital and during hospitalization. Duing the first 2 months, serum transaminases, bilirubin, prothrombin time, cholesterol and albumin, were checked weekly to detect any liver damage due to pravastatin. Serum creatine kinase activity was also checked every month. Tumour status was followed by US or computed tomography at least 4 times each year and tumour marker (AFP, alfa-fetoprotein) monthly. Maximal diameters of the main tumours in each group were sequentially measured with US at 2, 6 and 12 months after starting pravastatin to evaluate its effect on tumour growth.

### Measurement of urinary pravastatin

Compliance with pravastatin treatment was assessed by detecting pravastatin in urine according to the method described previously (Koga et al, 1995). Urine was obtained every 2 months after entry.

### Statistical analysis

Fisher's exact (two-tailed) test was used to compare the baseline characteristics of both groups. Survival curves were generated by the Kaplan–Meier method. The log-rank test was used to compare survival. Factors contributing to survival were selected using the Cox proportional-hazards regression analysis. Changes in laboratory profiles between the 2 groups were compared using Mann–Whitney test.

### RESULTS

91 patients with advanced HCC were enroled between February 1990 and January 1993. All patients underwent TAE and oral administration of 5-FU. 8 patients were withdrawn because of progressive liver dysfunction. The control ($n$ = 42) and pravastatin ($n$ = 41) groups were similar in terms of age, sex, liver function, renal function, stage of disease (The Liver Cancer Study Group of Japan, 1989), and presence of vascular invasion in portal veins, extra-hepatic spread or past history of encephalopathy (Table 1) at the start of pravastatin treatment. Both groups were not different in TAE-related complications. 33 patients in the pravastatin group had cirrhosis versus 34 controls. In the pravastatin group, the Child–Pugh classification in the cirrhotic patients was class A for 5 patients and class B for 28, compared with 4 and 30 in the control group, respectively. The Karnofsky performance scale was 80 to 90 for 34 patients and 60 to 70 for 7 patients in the pravastatin group versus 80 to 90 for 36 patients and 60 to 70 for 6 patients in the control group. HBs Ag was positive in 5 pravastatin patients and 4 controls. Anti-HCV antibody by second or third generation ELISA (Ortho Diagnostics, Tokyo) was positive in 33

© 2001 Cancer Research Campaign

**Table 1** Baseline demographics for pravastatin and control groups

| Variable | Pravastatin group (*n* = 41) *n* (%) | Control group (*n* = 42) *n* (%) | *P* value* |
|---|---|---|---|
| Age (y) | | | |
| <60 | 15 (37) | 19 (45) | >0.2 |
| ≥60 | 26 (63) | 23 (55) | |
| Sex | | | |
| Female | 10 (24) | 8 (20) | >0.2 |
| Male | 31 (76) | 34 (80) | |
| Tumour stage | | | |
| IV | 11 (27) | 13 (31) | >0.2 |
| II or III | 30 (73) | 29 (69) | |
| Vascular invasion in portal veins | | | |
| Yes | 5 (12) | 6 (14) | >0.2 |
| No | 36 (88) | 36 (86) | |
| Extra-hepatic spread | | | |
| Yes | 2 (5) | 1 (2) | >0.2 |
| No | 39 (95) | 41 (98) | |
| Serum ALT** level | | | |
| <60 U $l^{-1}$ | 25 (61) | 23 (55) | >0.2 |
| ≥60 U $l^{-1}$ | 16 (39) | 19 (45) | |
| Serum alkaline phosphatase level | | | |
| <200 IU $l^{-1}$ | 26 (63) | 24 (57) | >0.2 |
| ≥200 IU $l^{-1}$ | 15 (37) | 18 (43) | |
| Serum albumin level | | | |
| ≥35 g $l^{-1}$ | 21 (51) | 20 (47) | >0.2 |
| <35 g $l^{-1}$ | 20 (49) | 22 (53) | |
| Serum total bilirubin level | | | |
| <22 micro mol $l^{-1}$ | 17 (41) | 19 (45) | >0.2 |
| ≥22 micro mol $l^{-1}$ | 24 (59) | 23 (55) | |
| Serum creatinine level | | | |
| <1.2 mg $dl^{-1}$ | 21 (51) | 20 (47) | >0.2 |
| ≥1.2 mg $dl^{-1}$ | 20 (49) | 22 (53) | |
| Past history of encephalopathy | | | |
| Yes | 2 (5) | 2 (5) | >0.2 |
| No | 39 (95) | 40 (95) | |

Tumour stage was according to the criteria of the Liver Cancer Study Group of Japan (The Liver Cancer Study Group of Japan, 1989). *Fisher's exact test; **alanine aminotransferase.

of the pravastatin group and 35 of the control group. Patients were unresectable because of extensive tumour in 28 pravastatin patients and 30 controls and because of advanced liver disease in 13 and 12, respectively. The median follow-up was 11 months (range 2 to 66 months) by the end of February 1998. No patients were lost to follow-up.

The duration of pravastatin administration was 16.5 ± 9.8 months (mean ± SD). Pravastatin was discontinued in all patients because of advancing disease and not because of any adverse effects of the medicine. The reasons for discontinuation were hyperbilirubinaemia in 7 patients, prolonged prothrombin time in 5, massive ascites in 8, hyperammonaemia in 9, and hypoalbuminaemia in 12. No patient had an elevation of ALT levels to >150 U $l^{-1}$. During the administration period, pravastation was detected in all urine samples.

Serum ALT and total bilirubin did not differ between the 2 groups at 2 and 6 months after the start of pravastatin (Table 2). However, the serum cholesterol concentration was lower in the pravastatin group at 2 and 6 months ($P$ < 0.001 for both). The median of total bilirubin level 1 year after entry was 28 micro mol $l^{-1}$ ($n$ = 25) in the pravastatin group and 43 ($n$ = 11) in controls ($P$ = 0.04, Mann–Whitney test). In addition, the median of serum albumin level 1 year after entry was 31 g $l^{-1}$ in the pravastatin and 26 in the control group ($P$ = 0.02). These results suggested that liver function deteriorated more rapidly in the control group.

By the end of February 1998, all patients in both groups had died due to HCC progression and/or hepatic failure. The median survival was 18 months in the pravastatin group and 9 months in controls. Survival in the pravastatin group was significantly longer ($P$ = 0.006 by the log-rank test) (Figure 2). Using the Cox proportional-hazards model, pravastatin was a significant factor contributing to prolonged survival ($P$ = 0.02 for univariate analysis and $P$ = 0.005 for multivariate analysis) (Table 3). Absence of the vascular invasion in portal veins was also a significant factor contributing to prolonged survival ($P$ = 0.03 for multivariate analysis).

Serum AFP level was lower in the pravastatin group compared with controls 6 months and 1 year after the entry ($P$ = 0.04 and $P$ = 0.03, respectively; Mann–Whitney test) (Table 2). Regression of tumour was not observed in the pravastatin group at 2, 6 or 12 months. However, increase in maximal diameter was significantly less in the pravastatin group at 6 and 12 months compared with controls ($P$ = 0.03 and $P$ = 0.01, respectively; Mann–Whitney test), suggesting that tumour growth was suppressed by pravastatin.

In the control group, 35 patients died of tumour progression and 7 died of hepatic failure. In the pravastatin group, 36 died of tumour progression and 5 died of hepatic failure. Of the 36 patients who died of tumour progression in the pravastatin group, 1 had severe cholestasis due to tumour invasion into the bile ducts, 3 had massive pleural effusion due to lung metastasis, and 28 had

 *© 2001 Cancer Research Campaign*

**Table 2** Changes in liver function test and tumour markers after start of pravastatin administration

| Variable | Pravastatin group (*n* = 41) | Control group (*n* = 42) | *P* value |
|---|---|---|---|
| Serum ALT* level (U $l^{-1}$) | | | |
| Baseline | 58# (42–85)## | 63 (45–97) | > 0.2 |
| 2M | 57 (39–90) (*n* = 41) | 65 (42–92) (*n* = 42) | > 0.2 |
| 6M | 60 (40–86) (*n* = 35) | 59 (43–108) (*n* = 22) | > 0.2 |
| 1Y | 52 (36–74) (*n* = 25) | 68 (48–93) (*n* = 11) | > 0.2 |
| Serum total bilirubin level (micro mol $l^{-1}$) | | | |
| Baseline | 24 (18–37) | 25 (18–40) | > 0.2 |
| 2M | 25 (19–35) (*n* = 41) | 28 (19–44) (*n* = 42) | > 0.2 |
| 6M | 28 (21–38) (*n* = 35) | 36 (25–55) (*n* = 22) | > 0.2 |
| 1Y | 28 (22–40) (*n* = 25) | 43 (24–58) (*n* = 11) | 0.04 |
| Serum albumin level (g $l^{-1}$) | | | |
| Baseline | 35 (29–40) | 36 (26–38) | > 0.2 |
| 2M | 36 (28–41) (*n* = 41) | 34 (25–36) (*n* = 42) | > 0.2 |
| 6M | 33 (26–37) (*n* = 35) | 29 (24–34) (*n* = 22) | 0.06 |
| 1Y | 31 (24–35) (*n* = 25) | 26 (23–32) (*n* = 11) | 0.02 |
| Serum AFP level (ng $dl^{-1}$) | | | |
| Baseline | 130 (25–5140) | 120 (5–3450) | > 0.2 |
| 2M | 108 (21–4270) (*n* = 41) | 172 (45–6710) (*n* = 42) | > 0.2 |
| 6M | 218 (60–6540) (*n* = 35) | 292 (85–9573) (*n* = 22) | 0.04 |
| 1Y | 261 (78–7940) (*n* = 25) | 4140 (140–17300) (*n* = 11) | 0.03 |
| Serum total cholesterol level (mmol $l^{-1}$) | | | |
| Baseline | 4.30 (3.67–5.03) | 4.20 (3.58–4.96) | > 0.2 |
| 2M | 3.56 (3.25–3.94) (*n* = 41) | 3.93 (3.42–4.75) (*n* = 42) | < 0.001 |
| 6M | 3.24 (2.80–3.82) (*n* = 35) | 3.78 (3.52–4.63) (*n* = 22) | < 0.001 |
| 1Y | 3.10 (2.63–3.55) (*n* = 25) | 3.40 (2.86–4.07) (*n* = 11) | 0.03 |
| Diameter of main tumour (mm) | | | |
| Baseline | 38 (22–48) | 36 (24–52) | > 0.2 |
| 2M | 40 (24–52) (*n* = 41) | 43 (25–62) (*n* = 42) | > 0.2 |
| 6M | 45 (30–67) (*n* = 35) | 60 (34–83) (*n* = 22) | 0.03 |
| 1Y | 52 (42–78) (*n* = 25) | 73 (45–106) (*n* = 11) | 0.01 |

2M, 2 months after pravastatin; 6M, 6 months after pravastatin; 1Y, 1 year after pravastatin. # Median of values, ## range of values.

massive ascites due to the obstruction of the portal vein by tumour. Of the 5 patients who died of hepatic failure in the pravastatin group, 3 patients developed hepatic coma with progressive cirrhosis, and 2 had rupture of gastric varices.

### DISCUSSION

Chemotherapy that prolongs survival is not available for advanced HCC. To test whether pravastatin, a potent HMG-CoA reductase inhibitor, might increase the survival of patients with advanced HCC, we designed a randomized clinical trial with death as the primary endpoint. We chose TAE and 5-FU as standard treatment before introducing pravastatin administration. TAE prevents tumour progression (Charnsagavej et al, 1983; Yamada et al, 1983; Stefanini et al, 1995) and is one of the standard treatments for unresectable HCC in Japan. 5-FU also has activity against HCC (Cavalli et al, 1981; Coi et al, 1984; Falkson et al, 1984). To avoid any bias based on pretreatment, all 91 patients underwent a single TAE followed by oral 5-FU at a uniform dose for 2 months. 83 patients completed the standard treatment and were randomly assigned to pravastatin or control groups.

Patients in the pravastatin group survived significantly longer than those in the control group (Figure 2). Analysis using the Cox proportional-hazards model showed that treatment with pravastatin was the significant factor contributing to prolonged survival ($P$ = 0.02 for univariate analysis and $P$ = 0.005 for multivariate analysis). This result suggests that HMG-CoA reductase inhibitors offer a survival advantage in the treatment of advanced HCC.

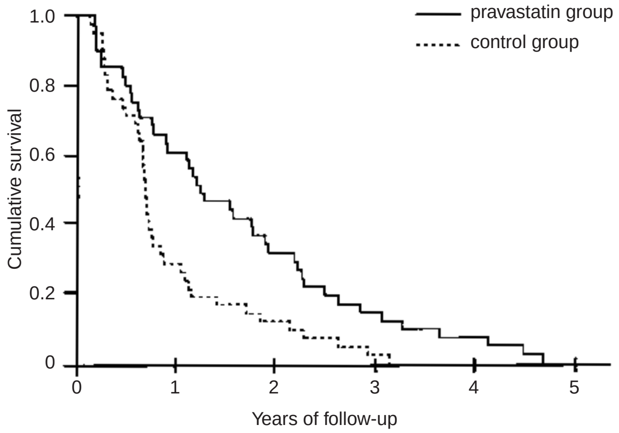

**Figure 2** Kaplan–Meier survival curves in pravastatin (*n* = 41) and control (*n* = 42) groups. The median survival was 18 months in the pravastatin group and 9 months in the control group ($P$ = 0.006 by the log-rank test)

The biochemical data after randomization suggested that liver function deteriorated more rapid in patients who were not taking pravastatin (Table 2). This may represent more rapid progression of either tumour or underlying liver disease in the control groups. Serum AFP concentrations more rapidly increased in controls (Table 2). Regression of the main tumour was not observed in the pravastatin group. Yet, growth of the main tumours was significantly slowed at 6 and 12 months after pravastatin administration, suggesting that pravastatin suppressed tumour growth. Pravastatin effects on survival may therefore have resulted from stabilization

© 2001 Cancer Research Campaign

**Table 3** Factors contributing to survival in HCC patients

| | Univariate analysis | | | Multivariate analysis | | |
|---|---|---|---|---|---|---|
| Variable | Risk ratio | 95% CI* | *P* value# | Risk ratio | 95% CI | *P* value |
| Age | | | | | | |
| < 60 | 1 | | | 1 | | |
| ≥ 60 | 1.72 | 0.58–4.84 | >0.2 | 1.30 | 0.67–2.65 | >0.2 |
| Sex | | | | | | |
| Female | 1 | | | 1 | | |
| Male | 0.62 | 0.22–1.72 | >0.2 | 0.82 | 0.38–1.70 | >0.2 |
| Tumour stage | | | | | | |
| IV | 1 | | | 1 | | |
| II or III | 0.67 | 0.22–2.06 | >0.2 | 0.72 | 0.34–1.63 | >0.2 |
| Vascular invasion in portal veins | | | | | | |
| Yes | 1 | | | 1 | | |
| No | 0.38 | 0.12–1.25 | 0.10 | 0.18 | 0.04–0.81 | 0.03 |
| Extra-hepatic spread | | | | | | |
| Yes | 1 | | | 1 | | |
| No | 0.65 | 0.22–1.98 | >0.2 | 0.57 | 0.25–1.62 | >0.2 |
| Serum ALT## level | | | | | | |
| <60 U $l^{-1}$ | 1 | | | 1 | | |
| ≥60 U $l^{-1}$ | 1.46 | 0.45–4.64 | >0.2 | 1.80 | 0.85–4.16 | >0.2 |
| Serum alkaline phosphatase level | | | | | | |
| <200 IU $l^{-1}$ | 1 | | | 1 | | |
| ≥200 IU $l^{-1}$ | 2.05 | 0.66–6.35 | >0.2 | 1.47 | 0.64–3.67 | >0.2 |
| Serum albumin level | | | | | | |
| ≥35 g $l^{-1}$ | 1 | | | 1 | | |
| <35 g $l^{-1}$ | 1.33 | 0.43–4.35 | >0.2 | 1.58 | 0.68–3.15 | >0.2 |
| Serum total bilirubin level | | | | | | |
| <22 micro mol $l^{-1}$ | 1 | | | 1 | | |
| >22 micro mol $l^{-1}$ | 2.3 | 0.71–7.46 | >0.2 | 1.35 | 0.62–3.05 | >0.2 |
| Serum creatinine level | | | | | | |
| <1.2 mg $dl^{-1}$ | 1 | | | 1 | | |
| ≥1.2 mg $dl^{-1}$ | 1.84 | 0.56–6.13 | >0.2 | 1.63 | 0.55–4.65 | >0.2 |
| Past history of encephalopathy | | | | | | |
| Yes | 1 | | | 1 | | |
| No | 0.67 | 0.21–2.08 | >0.2 | 0.52 | 0.18–1.50 | >0.2 |
| Pravastatin administration | | | | | | |
| No | 1 | | | 1 | | |
| Yes | 0.42 | 0.20–0.83 | 0.02 | 0.35 | 0.17–0.61 | 0.005 |

*CI denotes confidence interval. # Cox proportional-hazards regression analysis. ## analine aminotransferase.

of the tumour. Signal transduction inhibitors are generally cytostatic in their activity against malignant cells, and our results are consistent with other in vivo studies (Stebolt-Leopold et al, 1999).

Long-term administration of a daily dosage of 40 mg of pravastatin has been reported to prevent cardiovascular events (Byington et al, 1995; Shepherd et al, 1995). This dosage was well-tolerated without severe adverse effects. Because our patients had advanced HCC with chronic hepatitis and/or cirrhosis, we administered a daily dose of 20 mg of pravastatin during the first 2 weeks to check adverse effects. No significant problems were noted, and all patients subsequently received 40 mg of pravastatin. During the first 2 months, we found no elevations of serum bilirubin or transaminase values which could be attributed to pravastatin. Throughout the observation period, there were no significant differences in liver function tests or haematologic data between the 2 groups. After 2 months, pravastatin was continued until exclusion criteria were satisfied. All patients in the pravastatin group appeared to die of progressive disease.

Patients with chronic liver disease often have muscle cramps. In this study, the frequency of muscle cramps did not differ between the 2 groups (data not shown). None of the patients in either group showed more than a 10-fold elevation in serum creatine kinase values. In general, patients with advanced HCC tolerated long-term administration of a daily dose of 40 mg of pravastatin.

HMG-CoA reductase inhibitors have been reported to have cytostatic activity, possibly due to suppression of protein isoprenylation (Sinensky et al, 1990). Cholesterol is a primary source for membrane formation and thus is in great demand in rapid-growth tissues such as cancers. Previously, we reported that HMG-CoA reductase activity and protein content, as well as cholesterol biosynthesis, were increased in human HCC tissues (Kawata et al, 1990). The liver has a high affinity for pravastatin (Tsujita et al, 1986). Prior to this study, we observed that pravastatin at a daily dose of 40 mg led to a significant decrease in serum concentrations of cholesterol and AFP in 3 patients with hypercholesterolaemia associated with HCC as a paraneoplastic syndrome (data not shown). This agent might have been effectively taken up to hepatoma cells in vivo. In future studies, to clarify the mechanism(s) whereby pravastatin may prolong survival in advanced HCC, liver biopsy should be done before and after therapy to monitor changes in protein isoprenylation and cholesterol content in tumour cells.

This study was not blinded, although it was randomized. The study also included treatment with TAE and oral 5-FU, although randomization was done after completion of those treatments.

 *© 2001 Cancer Research Campaign*

These factors may have introduced bias in assessing the effect of pravastatin on survival. In the future, a randomized, placebo-controlled, double-blind study will be needed to clarify whether HMG-CoA reductase inhibitors are useful as adjuvant anti-cancer therapy.

In conclusion, pravastatin prolonged the survival of patients with advanced HCC. This result should encourage the development of HMG-CoA reductase inhibitors as adjuvant therapy against HCC.

### ACKNOWLEDGEMENTS

This work was supported in part by a Grant-in-Aid for Cancer Research to S Kawata (Grant No. 07274240) from the Ministry of Education, Science, and Culture in Japan.

### REFERENCES

Byington KP, Jukema JW, Salonen JT, Bertram P, Bruscheke AV, Hoen H, Furberg CD and Mancini GBJ (1995) Reduction in cardiovascular events during pravastatin therapy. Pooled analysis of clinical events of the pravastatin atherosclerosis intervention program. *Circulation* **92**: 2419–2425

Cavalli F, Rozencweig M, Goldhirsch A and Hansen HH (1981) Phase II study of oral VP-16-213 in hepatocellular carcinoma. *Eur J Cancer Clin Oncol* **17**: 1079–1082

Charnsagavej S, Chuang VP, Wallace S, Soo CS and Bowers T (1983) Transcatheter management of promary carcinoma of the liver. *Radiology* **147**: 51–55

Coi TK, Lee NW and Wong J (1984) Chemotherapy for advanced hepatocellular carcinoma. Adriamycin versus quadruple chemotherapy. *Cancer* **53**: 401

Di Bisceglie AM (1995) Hepatitis C and hepatoecllular carcinoma. *Seminar Liver Dis* **15**: 64–69

El-Serag HB and Mason AC (1999) Rising incidence of heptocellular carcinoma in the United States. *N Engl J Med* **340**: 745–750

Falkson G, MacIntyre JM, Moertel CG, Johnson LA and Scherman RC (1984) Primary liver cancer: An Eastern JM Cooperative Oncology Group Trial. *Cancer* **54**: 970–977

Gibbs JB, Pompliano DL, Mosser SD, Rands E, Lingham RB, Singh SB, Scolnick EM, Kohl NE and Oliff A (1993) Selective inhibition of farnesyl: transferase blocks ras processing in vivo. *J Biol Chem* **268**: 7617–7620

Goldstein JL and Brown MS (1990) Regulation of the mevalonate pathway. *Nature* **343**: 425–430

Goldstein JL, Helgeson JAS and Brown MS (1979) Inhibition of cholesterol synthesis with compactin renders growth of cultured cells dependent on the low density lipoprotein receptors. *J Biol Chem* **254**: 5403–5409

Habenicht AJR, Glomset JA and Ross R (1980) Relation of cholesterol and mevalonic acid to the cell cycle in smooth muscle and Swiss 3T3 cells stimulated to divide by platelet-derived growth factor. *J Biol Chem* **255**: 5134–5140

James GL, Goldstein JL, Brown MS, Rawson TE, Somers TC, McDowell RS, Crowley CW, Lucas BK, Levinson AD and Marsters Jr JC (1993) Benzodiazepine peptidomimetics: protein inhibitor of ras farnesylation in animal cells. *Science* **260**: 1937–1942

Kandutsch AA and Chen HW (1979) Consequences of blocked sterol synthesis in cultured cells, DNA synthesis and membrane composition. *J Biol Chem* **252**: 409–415

Kawata S, Takaishi K, Nagase T, Ito N, Tamura S, Matsuzawa Y and Tarui S (1990) Increase in the active form of 3-hydroxy-3-methylglutaryl coenzyme A reductase in human hepatocellular carcinoma: Possible mechanism for alteration of cholesterol biosynthesis. *Cancer Res* **50**: 3270–3273

Koga T, Kawabata K, Arai K, Matsushima N, Koike H, Komai T, Rei M and Nakamura H (1995) Comparative pharmacokinetics of pravastatin and simvastatin. *Bull Mol Biol Med* **20**: 103–105

Kohl NE, Mosser SD, deSolms SJ, Giuliani EA, Pompliano DL, Graham SL, Smith RL, Scolnick EM, Oliff A and Gibbs JB (1993) Selective inhibition of ras-dependent transformation by a farnesyltransferase inhibitor. *Science* **260**: 1934–1937

Maltese WA, Defendini R, Green RA, Sheridan KM and Donley DK (1985) Suppression of murine neuroblastoma growth in vivo by mevinolin, a competitive inhibitor of 3-hydroxy-3-methylglutaryl coenzyme A reductase. *J Clin Invest* **76**: 1748–1754

Matsuda Y, Kawata S, Nagase T, Maeda Y, Yamasaki E, Kiso S, Ishiguro H and Matsuzawa Y (1994) Interleukin-6 in transcatheter arterial embolization for patients with hepatocellular carcinoma. *Cancer* **73**: 53–57

Okuda K (1992) Hepatocellular carcinoma: recent progress. *Hepatology* **15**: 948–963

Okuda K, Fujimoto I, Hanai A and Urano Y (1987) Changing incidence of hepatocellular carcinoma in Japan. *Cancer Res* **47**: 4967–4972

Shepherd J, Lobbe SM, Ford I, Isles CG, Lorimer AR, Macfarlane PW, Mackillop JH, Packard CJ, for the West of Scotland Coronary Prevention Study Group (1995) Prevention of coronary heart disease with pravastatin in men with hypercholesterolemia. *N Engl J Med* **333**: 1301–1307

Simonetti RG, Camma C, Fiorello F, Politi F, D'amico G and Pagliaro L (1991) Hepatocellular carcinoma. A worldwide problem and the major risk factors. *Dig Dis Sci* **36**: 962–972

Sinensky M, Beck LA, Leonard S and Evans R (1990) Differential inhibitory effects of lovastatin on protein isoprenylation and sterol synthesis. *J Biol Chem* **265**: 19937–19941

Stebolt-Leopold JS, Dudley DT, Herrera R, Becelaere KV, Wiland A, Gowan RC, Tecle H, Barrett SD, Bridges A, Przybranowski S, Leopold WR and Saltiel AR (1999) Blockade of the MAP kinase pathway suppresses growth of colon tumors in vivo. *Nature Med* **5**: 810–816

Stefanini GF, Amorati P, Beselli M, Mucci F, Celi A, Arienti V, Roversi R, Rossi C, Re G and Gasbarrini G (1995) Efficacy of transarterial targeted treatment on survival of patients with hepatocellular carcinoma. *Cancer* **75**: 2427–2434

The Liver Cancer Study Group of Japan (1989) The general rules of the clinical and pathological study of primary liver cancer. *Jpn J Sur* **19**: 98–129

Tsujita Y, Kuroda M, Shimada Y, Tanzawa K, Arai M, Kaneko I, Tanaka M, Matsuda H, Tarumi C, Watanabe Y and Fujii S (1986) CS-514, a competitive inhibitor of 3-hydroxy-3-methylglutaryl coenzyme A reductase: tissue-selective inhibiton of sterol synthesis and hypolipidemic effect on various animal species. *Biophys Biochem Acta* **877**: 50–60

Yamada R, Sato M, Kawabata M, Nakatsuka H, Nakamura K and Takashima S (1983) Hepatic artery embolization in 120 patients with unresectable hepatoma. *Radiology* **148**: 397–401

### APPENDIX

### Current author addresses

Dr Kawata: Second Department of Internal Medicine, Yamagata University School of Medicine, 2-2-2 Iida-Nishi, Yamagata 990-9585, Japan.

Drs Yamasaki, Nagase, Inui, Ito, Tamura, and Matsuzawa: Department of Internal Medicine and Molecular Science, Graduate School of Medicine, B5, Osaka University, 2-2 Yamada-oka, Suita, Osaka 565-0871, Japan.

Drs Matsuda and Inada: Department of Internal Medicine, Toyonaka Municipal Hospital, 4-14-1 Shibahara-cho, Toyonaka, Osaka 5675, Japan.

Dr Noda: Department of Internal Medicine, Suita Municipal Hospital, 2-13-20 Katayama-cho, Suita, Osaka 564, Japan.

Dr. Imai: Department of Internal Medicine, Ikeda Municipal Hospital, 3-1-18 Johnan, Ikeda, Osaka 563; Japan.

© 2001 Cancer Research Campaign
